# Understanding and preventing mitochondrial oxidative damage

**DOI:** 10.1042/BST20160108

**Published:** 2016-10-19

**Authors:** Michael P. Murphy

**Affiliations:** MRC Mitochondrial Biology Unit, Hills Road, Cambridge CB2 0XY, U.K.

**Keywords:** mitochondria, oxidative damage, ROS

## Abstract

Mitochondrial oxidative damage has long been known to contribute to damage in conditions such as ischaemia–reperfusion (IR) injury in heart attack. Over the past years, we have developed a series of mitochondria-targeted compounds designed to ameliorate or determine how this damage occurs. I will outline some of this work, from MitoQ to the mitochondria-targeted *S*-nitrosating agent, called MitoSNO, that we showed was effective in preventing reactive oxygen species (ROS) formation in IR injury with therapeutic implications. In addition, the protection by this compound suggested that ROS production in IR injury was mainly coming from complex I. This led us to investigate the mechanism of the ROS production and using a metabolomic approach, we found that the ROS production in IR injury came from the accumulation of succinate during ischaemia that then drove mitochondrial ROS production by reverse electron transport at complex I during reperfusion. This surprising mechanism led us to develop further new therapeutic approaches to have an impact on the damage that mitochondrial ROS do in pathology and also to explore how mitochondrial ROS can act as redox signals. I will discuss how these approaches have led to a better understanding of mitochondrial oxidative damage in pathology and also to the development of new therapeutic strategies.

## Introduction

Mitochondria are at the heart of metabolism, supplying most of the cell's ATP, housing a myriad of other metabolic networks and contributing to both apoptotic and necrotic cell death. Therefore, it is important to know how mitochondria work and how the changes in factors such as reactive oxygen species (ROS) damage mitochondria and thereby lead to pathology. There is also a need to develop drugs to prevent the mitochondrial dysfunction that occurs in a range of diseases [1]. One way of achieving these goals is to make molecules targeted to mitochondria that can act as probes, or as drugs to decrease mitochondrial damage. To do this, my lab, along with many collaborators and colleagues, have developed methods to target molecules to mitochondria by conjugation to lipophilic cations, and here the ways in which this can be done will be discussed.

## Targeting small molecules to mitochondria using the triphenylphosphonium lipophilic cation

**Figure 1.**
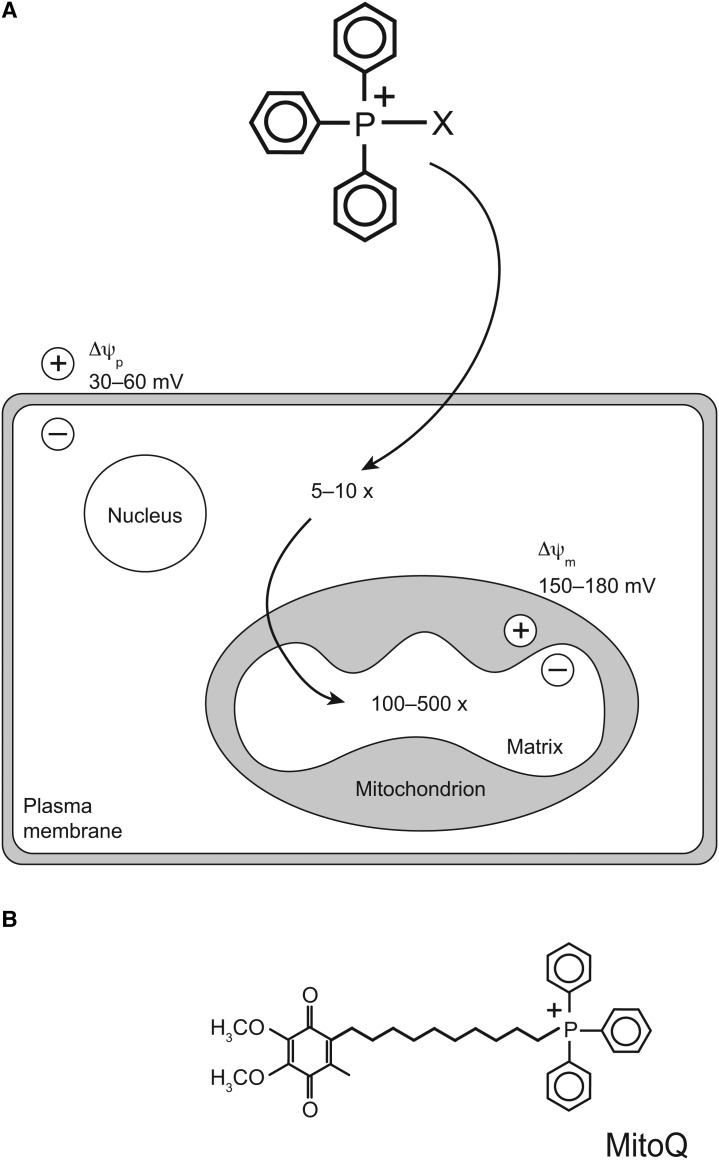
Uptake of TPP compounds by mitochondria. (**A**) A TPP molecule attached to a moiety to be delivered to mitochondria (X), is shown being accumulated, driven by the plasma (Δψ_p_) and mitochondrial (Δψ_m_) membrane potentials. (**B**) Structure of the mitochondria-targeted antioxidant MitoQ.

The most widely used approach to direct small molecules to mitochondria *in vivo* is by conjugation to a lipophilic cation [2,3] (Figure 1A). The charge is spread over a large, hydrophobic surface area, allowing these cations to pass easily through membranes and accumulate in the mitochondrial matrix in response to the membrane potential [3]. The Nernst equation indicates that the uptake of singly charged cations increases 10-fold for every 61.5 mV of membrane potential at 37°C [3]; therefore, lipophilic cations should concentrate several hundred-fold greater in mitochondria (Figure 1A). The lipophilic triphenylphosphonium cation (TPP) has been the most widely used to direct a range of moieties to mitochondria *in vivo*, including redox probes, antioxidants and spin traps [2,4,5]. The TPP moiety is relatively easy to introduce into a compound by displacing a leaving group by reaction with triphenylphosphine [6]. TPP cations are accumulated by mitochondria within organs such as the heart, liver and kidneys *in vivo* following oral, intravenous or intraperitoneal delivery consistent with their uptake from the circulation driven by the plasma and mitochondrial membrane potentials [6–8]. Therefore, the TPP moiety has been widely used to target many molecules to mitochondria, both as probes and as potential therapies. In the following section, I consider the development of mitochondria-targeted antioxidants.

## Mitochondria-targeted antioxidants

Mitochondria are a major source of ROS and are also easily damaged by ROS [9]. This mitochondrial oxidative damage contributes to dysfunction and cell death in a range of diseases [9]. Therefore, there has been an interest in developing mitochondria-targeted antioxidants designed to ameliorate mitochondrial oxidative damage [2,10]. The rationale for the development of mitochondria-targeted antioxidants is that although oxidative damage to mitochondria contributes to a wide range of pathologies, antioxidant therapies have performed poorly in clinical trials [11,12]. As discussed in the extensive critical summary of clinical trials to date [11], trials of many of the most common antioxidants, such as vitamin E and vitamin C, showed no benefit to patients. Failures such as this could be because oxidative damage is not a major contributor to disease. Alternatively, the lack of success may be because of the small proportion of the antioxidant *in vivo* actually located in the mitochondria, where it is needed most to counteract mitochondrial oxidative damage. Mitochondria-targeted antioxidants were developed to overcome this targeting limitation [12].

Many mitochondria-targeted antioxidants have been developed by conjugation to the TPP cation, most of which have shown protection against oxidative damage in mitochondria and cells, although only a few have been used *in vivo*. In animal studies, the most widely used has been MitoQ (Figure 1B), which is a ubiquinone moiety linked to a TPP cation by a 10-carbon alkyl chain [2,5]. The ubiquinol form of MitoQ acts as an antioxidant becoming oxidized to a ubiquinone, which is then reduced by complex II back to ubiquinol, restoring its antioxidant efficacy [13]. As MitoQ is primarily found adsorbed to the mitochondrial inner membrane, and its linker chain enables the active ubiquinol antioxidant component to penetrate deeply into the membrane core, it is an effective antioxidant against lipid peroxidation [14]. The oral administration of MitoQ to rodents is safe [8] and *in vivo* studies have shown that MitoQ can protect against oxidative damage in many animal models of pathology, including cardiac ischaemia–reperfusion (IR) injury [15], hypertension [16], sepsis [17,18], kidney damage in type I diabetes [19], MPTP toxicity in the brain [20] and kidney cold preservation for organ transplantation [21]. Many other mitochondria-targeted antioxidants, in addition to MitoQ, have since been developed such as SkQ [3]. Therefore, antioxidants targeted to mitochondria such as MitoQ are protective against pathological changes in animal models of human diseases.

The results in animal models led to the assessment of MitoQ in a human phase II trial in Parkinson's disease, the PROTECT trial (www.clinicaltrials.gov, NCT00329056) [22]. Although MitoQ showed no difference from placebo [22], this work did show that MitoQ can be safely administered to patients for a year. A second small human trial with MitoQ, the CLEAR trial on patients with chronic hepatitis C virus [23] (www.clinicaltrials.gov, NCT00433108), showed a decrease in markers of liver damage and was the first report of a clinical benefit from mitochondrial-targeted antioxidants in humans. Although future work is required, these findings suggest that antioxidants targeted to mitochondria may be applicable to human pathologies involving mitochondrial oxidative damage.

## Targeting mass spectrometric ROS probes to mitochondria

It is often important to measure ROS levels. In cells, changes in specific ROS such as superoxide can be inferred from the changes in fluorescence of probes such as hydroethidine [24] or MitoSOX [25], or for hydrogen peroxide with boronic acid-conjugated fluorophores [26]. Another approach is to utilize engineered proteins derived from green fluorescent protein (GFP) such as redox-sensitive GFP or HyPer [27,28]. These approaches produce robust and useful information, provided artefactual effects are recognized [29]. However, extension of these approaches from cells to living organisms is challenging. In some circumstances, optical techniques can be used, for example, by the use of two-photon microscopy [30] or through the use of bioluminescent probes [31]. In general, though, it is difficult to measure the levels of small, reactive molecules within living organisms.

Changes in ROS *in vivo* are often proposed to mediate damage and redox signals, but we do not have the techniques available to test these hypotheses properly [32]. One method to assess the levels of ROS *in vivo* is by the use of ‘exomarkers’. This approach has parallels with the use of biomarkers whereby changes in the levels of products, such as F_2_-isoprostanes, from the interaction of reactive species with endogenous molecules are used to infer changes in reactive species *in vivo* [33]. However, exomarkers differ in that an exogenous probe is administered to the organism. Within the living organism, the probe is modified by reactive species to generate an exomarker, diagnostic of the reactive species that can then be used to infer levels of reactive species *in vivo*. This approach has been used in the past, for example, in the selective reactivity of spin traps with free radicals [34].

**Figure 2.**
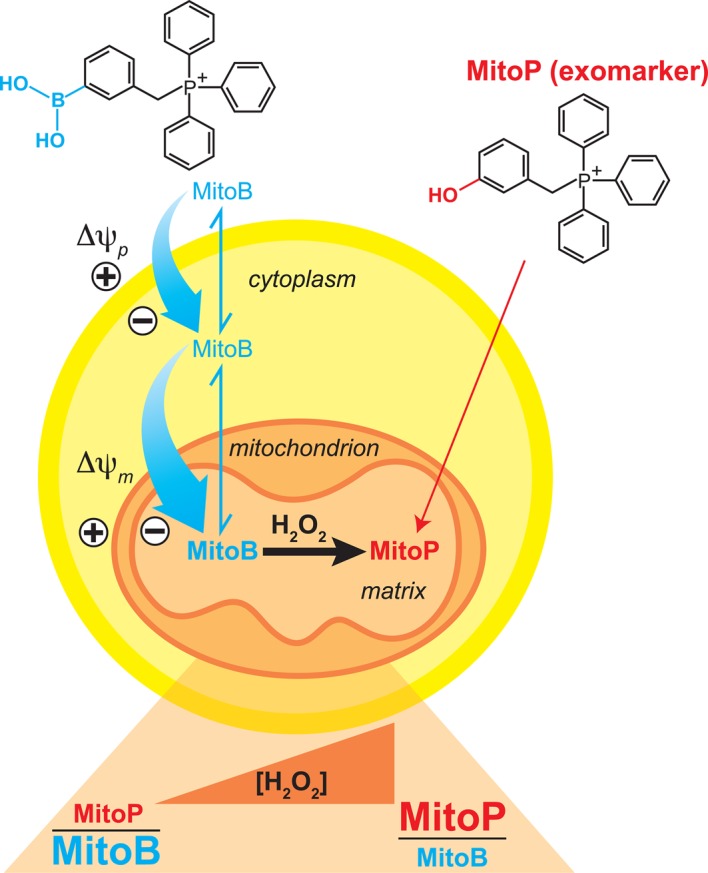
Using MitoB to assess mitochondrial hydrogen peroxide formation *in vivo.* Administration of MitoB to an animal model leads to the rapid accumulation of MitoB within cells and from there into mitochondria, driven by the plasma (Δψ_p_) and mitochondrial (Δψ_m_) membrane potentials. There MitoB will react with the local concentration of hydrogen peroxide to form MitoP causing the ratio of MitoP/MitoB to increase.

A focus on our work is to better understand how mitochondrial ROS such as hydrogen peroxide contribute to biological damage and redox signalling [9]. To address this unmet need, we developed an exomarker approach using a mitochondria-targeted mass spectrometric probe called 3-(dihydroxyboronyl)benzyltriphenylphosphonium bromide (MitoB) [35]. MitoB comprises the TPP moiety linked to an arylboronic acid [35] (Figure 2). The TPP moiety enables the molecules to pass rapidly through biological membranes to be rapidly cleared from the blood and to accumulate within the mitochondria driven by the large mitochondrial membrane potential. The arylboronic acid moiety attached to the TPP reacts directly but slowly with hydrogen peroxide to form a phenol without consuming a significant amount of hydrogen peroxide [26]. Kalyanaraman and colleagues [36] have since shown that arylboronates also react with peroxynitrite and hypohalous acids to generate the corresponding phenol. Therefore, the arylboronic acid moiety is a useful hydrogen peroxide-selective probe, provided that peroxynitrite and hypohalous acid production could be estimated independently. MitoB should accumulate rapidly within mitochondria *in vivo* and there be converted to the phenol, (3-hydroxybenzyl)triphenylphosphonium bromide (MitoP), in proportion to the local hydrogen peroxide concentration [35]. Therefore, measurements of the accumulation of the exomarker MitoP relative to the probe MitoB should indicate the relative changes in the average, local hydrogen peroxide concentration within mitochondria over the duration of the experiment.

Experiments are carried out by injecting MitoB into a living organism [35,37]. This results in the rapid delivery of MitoB to the mitochondria within the tissues and the animal is then allowed to resume normal behaviour for 3–6 h. Over time, the ratio of MitoP/MitoB in a tissue will increase due to the conversion of MitoB to MitoP. By expressing the results as the ratio of MitoP/MitoB over time, the assay corrects for changes in the uptake of MitoB into mitochondria in the tissue under consideration. The amount of MitoP relative to MitoB in tissue samples is measured by liquid chromatography–tandem mass spectrometry, relative to deuterated internal standards [35]. This approach was chosen because the TPP cation contains a fixed positive charge that greatly enhances the sensitivity of detection by mass spectrometry. MitoB has been used to assess changes in the production of mitochondrial hydrogen peroxide in *Drosophila* with age [35]. MitoB was also applied to mammalian systems and it was found that an intravenous injection of MitoB into a mouse enabled sufficient MitoB to be taken up into the heart to assess changes in mitochondrial ROS production during IR injury [37]. Therefore, MitoB can be used as a probe to assess mitochondrial hydrogen peroxide production *in vivo*, through generation of MitoP as an exomarker, and many other experimental models are currently being interrogated using MitoB and new types of probe designed to assess other aspects of mitochondrial function are also being investigated [38–40].

## Mitochondrial superoxide production during IR injury

IR injury occurs when the blood supply to a tissue is blocked for minutes to hours (ischaemia), and then restored (reperfusion). There has long been an interest in better understanding IR injury and in developing therapies to prevent its damaging effects. Ischaemic cells will die if blood flow is not restored, but it is during reperfusion itself that most IR damage is initiated. The first damaging event upon reperfusion is a burst of ROS production from mitochondria that initiates the pathology that develops over the minutes, days and weeks following reperfusion [41,42]. The initial burst of ROS production upon reperfusion directly causes oxidative damage to mitochondria, and in conjunction with dysregulation of calcium levels, this elevated ROS can also lead to induction of the mitochondrial permeability transition and on to cell death following reperfusion [41,43].

During ischaemia, the respiratory chain, redox active enzymes and electron carrier pools such as NADH and CoenzymeQ (CoQ) become maximally reduced. In parallel, mitochondria become progressively compromised due to the factors such as ATP depletion, lack of ion homeostasis, calcium overload and changes in pH [44]. Consequently, when ischaemic tissue is reperfused with oxygenated blood, the tacit assumption has been that inappropriately reduced and damaged mitochondrial components will spill electrons onto oxygen from numerous sites to form superoxide. While this model has appeal, there is now a large amount of evidence that points to respiratory complex I as the major source of mitochondrial superoxide upon reperfusion by reverse electron transport (RET) [41]. RET occurs when electrons are forced backwards through complex I. In forward transport, two electrons transfer from NADH to CoQ pumping four protons across the mitochondrial inner membrane to maintain the proton motive force (Δ*p*). Forward transport occurs when the redox driving force, the difference in reduction potentials (Δ*E*_h_), is greater than the energy required to pump protons across the inner membrane against the proton motive force (Δ*p*): 2Δ*E*_h_ > 4Δ*p*. However, the direction of electron transport can be reversed if Δ*p* is high and/or the CoQ pool is reduced, so that 4Δ*p* > 2Δ*E*_h_ [9,41]. If these conditions are met, electrons flow backwards through complex I and onto the flavin mononucleotide from where they can reduce NAD^+^ to NADH and also drive superoxide formation. Consistent with RET at complex I driving IR injury, many inhibitors of complex I are protective against IR injury [41].

For RET from complex I to occur during IR, the CoQ pool must be maintained highly reduced, so as to generate a near maximal Δ*p* by complexes III and IV, while also donating electrons for RET through complex I. Recently, our group showed that succinate accumulated to a far greater extent than any other mitochondrial metabolite as a terminal electron sink during ischaemia, thereby acting as an electron store that was then used to drive superoxide formation from complex I by RET during reperfusion [45]. *In vivo*, the succinate that accumulates over time in ischaemic tissues is very rapidly oxidized upon reperfusion back to baseline levels within 5 min [45]. This prompt clearance of succinate is likely due to its rapid oxidation in the mitochondrial matrix, sustained by the uptake of cytosolic succinate and is required for RET-driven ROS and IR injury, as was shown by manipulations that prevent the accumulation of succinate during ischaemia [45].

As we suggest that superoxide generation by RET at complex I upon reperfusion is the major source of IR injury, it is of particular relevance that complex I RET is dramatically altered by a structural change known as the active/deactive transition [46]. When complex I is not oxidizing NADH and pumping protons across the mitochondrial inner membrane, it gradually converts into a ‘deactive’ state, which is characterized by a conformational transition [46]. The physiological role of the active/deactive transition is unclear, but in the context of IR injury the most interesting aspect is that complex I readily undergoes deactivation during ischaemia with a half-life for deactivation of approximately 10–12 [47]. Upon reperfusion, complex I is rapidly reactivated and can support superoxide production by RET [47].

The active/deactive transition of complex I provides an appealing explanation for many therapeutic interventions that decrease IR injury. Deactive complex I exposes a critical cysteine residue (Cys39 in the ND3 subunit in mammals) that is occluded in active complex I [41,47]. Covalent modification of Cys39 locks complex I in the deactive state, and reversible modification of this cysteine residue by thiol reactive agents also temporarily locks complex I in the deactive state *in vivo*, thereby preventing ROS production by RET [41]. The overall effect is that electron flow through complex I is temporarily prevented upon reperfusion, thereby blocking superoxide production by RET during the crucial first few minutes of reperfusion. As the modification is reversible, complex I returns to full activity a few minutes after reperfusion, by which time the succinate accumulated during ischaemia will have been oxidized. This modification was demonstrated using the mitochondria-targeted *S*-nitrosothiol MitoSNO, and underlies the protective action of *S*-nitrosating agents such as MitoSNO against IR injury [37,41]. Together these findings have led us to propose a unifying framework that can be tested in future experiments while also suggesting approaches for the future development of targeted therapies against the generation of mitochondrial ROS in IR and related pathologies [41].

## Conclusion

The work outlined here shows that mitochondrial ROS production and oxidative damage are central to a range of pathologies. Through the ability to target small molecules to mitochondria *in vivo* by conjugation to lipophilic cations, we have been able to make some progress not only in the development of methods to assess mitochondrial ROS production *in vivo*, but also in the generation of new therapeutic strategies and potential drugs. While these approaches to understanding and intervening in mitochondrial oxidative damage are still at an early stage of development, the hope is that they will enable us to address better many of the diseases that affect our ageing populations.

## Abbreviations

CoQ, coenzymeQ; GFP, green fluorescent protein; IR, ischaemia–reperfusion; MitoB, 3-(dihydroxyboronyl)benzyltriphenylphosphonium bromide; MitoP, (3-hydroxybenzyl)triphenylphosphonium bromide; RET, reverse electron transport; ROS, reactive oxygen species; SOD, superoxide dismutase; TPMP, methyltriphenylphosphonium; TPP, triphenylphosphonium cation; Δ*p*, proton motive force.

## Funding

Work in the author's laboratory is supported by the Medical Research Council (U.K.) [MC_U105663142].
